# Pre-operative habitual dietary fibre stratifies 12-week immune–inflammatory recovery after oesophagectomy: a multicentre prospective cohort study

**DOI:** 10.3389/fnut.2025.1647811

**Published:** 2025-10-23

**Authors:** Yang Li, Yuan Chen, You Guo, Ling Wu, Hu Sun, Danqiong Wang, Tao Li, Na An, Jingfang Yan

**Affiliations:** ^1^Department of Geriatric Medicine, Shanxi Bethune Hospital, Shanxi Academy of Medical Sciences, Third Hospital of Shanxi Medical University, Tongji Shanxi Hospital, Taiyuan, China; ^2^Department of Geriatrics, Tongji Hospital, Tongji Medical College, Huazhong University of Science and Technology, Wuhan, China; ^3^Department of General Medicine, Xinzhou People’s Hospital, Xinzhou, China; ^4^Tumor Center, Shanxi Bethune Hospital, Shanxi Academy of Medical Sciences, Third Hospital of Shanxi Medical University, Tongji Shanxi Hospital, Taiyuan, China; ^5^Department of General Medicine, Shanxi Province Cancer Hospital/ Shanxi Hospital Affiliated to Cancer Hospital, Chinese Academy of Medical Sciences/Cancer Hospital Affiliated to Shanxi Medical University, Taiyuan, China

**Keywords:** oesophageal cancer, oesophagectomy, dietary fibre, pre-operative nutrition, prehabilitation, gut microbiota, short-chain fatty acids

## Abstract

**Objectives:**

To test whether habitual pre-operative dietary fibre predicts 12-week immune–inflammatory recovery after oesophagectomy.

**Methods:**

We conducted a multicentre prospective cohort across three tertiary hospitals. Adults with resectable oesophageal cancer completed a validated FFQ; total fibre (energy-adjusted) was grouped into sex-specific quartiles. Prespecified week-12 endpoints were: (i) a favourable inflammatory profile (CRP within reference or ≥50% fall plus NLR ≤ 3.0) and (ii) lymphocyte recovery (≥30% rise or ≥1.5 × 10^9·L^−1^). Robust Poisson models (clustered by site) estimated adjusted relative risks; dose–response was expressed per 10 g·day^−1^. Longitudinal biomarker trajectories (baseline→post-operative day 7 → week 12) used mixed-effects models.

**Results:**

Among 312 participants, event rates increased monotonically with higher fibre. Versus Q1, adjusted RRs for the favourable inflammatory profile were 2.36 (1.85–3.01) in Q3 and 2.62 (2.20–3.12) in Q4; for lymphocyte recovery, 1.63 (1.39–1.92) and 1.79 (1.65–1.95), respectively. Each +10 g·day^−1^ of fibre associated with RR 1.56 (1.34–1.82) for the favourable profile and 1.30 (1.17–1.45) for lymphocyte recovery. CRP and NLR declined more steeply and lymphocyte counts rose more in higher-fibre groups (time×quartile *p* = 1.68 × 10^−4^; 1.21 × 10^−4^; 2.26 × 10^−10^). Early infections and 30-day mortality did not differ convincingly (per-10 g RR 0.84, *p* = 0.333; overall 1.0%). FFQ–record ICC for fibre was 0.87.

**Conclusion:**

Higher habitual fibre before surgery was associated with materially better week-12 immune–inflammatory recovery after oesophagectomy, with consistent dose–response and longitudinal signals across centres. Routine pre-operative fibre appraisal offers a low-cost, clinic-ready stratifier for counselling and prehabilitation; interventional studies should test whether augmenting habitual fibre improves recovery trajectories.

## 1 Introduction

Oesophageal cancer remains a high-burden malignancy in which surgical cure is often achieved at the cost of profound peri-operative physiological stress. Beyond operative technique and enhanced-recovery protocols, the field still lacks low-cost, clinic-ready exposures that are legible to routine workflows yet meaningfully index the immune–inflammatory milieu that shapes convalescence (1–4). Habitual dietary fibre is a compelling candidate: it is measurable in minutes, modifiable in prehabilitation windows, and mechanistically linked to host inflammatory tone through the gut-microbiome’s short-chain-fatty-acid (SCFA) circuitry (5–8). In settings where oesophagectomy is common and nutrition counselling variably implemented, a fibre-centred, microbiome-informed lens offers a practical “pre-op phenotype” that can be elicited before admission and carried forward to outpatient recovery.

Multiple mechanistic and epidemiologic threads converge on a biologically coherent fibre → SCFA immunometabolic axis relevant to surgical recovery and tumour-adjacent inflammation. Fermentation of complex polysaccharides increases colonic pools of acetate, propionate and butyrate, metabolites that signal via nutrient-sensing G-protein–coupled receptors and histone-deacetylase inhibition to recalibrate innate immune tone, temper neutrophil-dominant responses, support epithelial-barrier integrity, and shape cytokine programs that favour resolution. Within a peri-operative context, such shifts plausibly steepen CRP/NLR recovery trajectories and facilitate lymphocyte reconstitution after major surgery (9–12).

A strength of focusing on habitual fibre is its standardisation and transportability across care settings. A validated, semi-quantitative food-frequency questionnaire (FFQ) can capture three-month intake with energy adjustment and allow categorisation into sex-specific quartiles (with ≥30 g·day^−1^ explored in sensitivity analyses), yielding a parsimonious exposure aligned with routine counselling (13–16). On the outcome side, routinely available venous markers—C-reactive protein (CRP), neutrophil-to-lymphocyte ratio (NLR), and absolute lymphocyte count—summarise systemic inflammatory tone and immune repopulation in ways that are interpretable on rounds and in clinics; embedding these measures at harmonised time-points (baseline pre-op, POD 7, and a clinically salient week-12 review) preserves clinical legibility while targeting an endpoint that matters for symptoms, complications and readiness for adjuvant care.

Accordingly, we conducted a multicentre, prospective cohort across three tertiary hospitals, ascertained pre-operative habitual dietary fibre via a validated FFQ, and followed immune–inflammatory markers from baseline→POD 7 → week 12 using prespecified, clinically legible week-12 endpoints that marry inflammatory tone (CRP/NLR) with immune repopulation (lymphocyte recovery). We analysed fibre both categorically and continuously with robust models accounting for hospital clustering and biologically grounded covariates, aiming for a validation-first, transportable workflow. We hypothesised that higher habitual pre-operative fibre would associate with a materially greater probability of a favourable inflammatory profile and lymphocyte recovery at week 12, alongside steeper post-operative CRP/NLR declines.

## 2 Methods

### 2.1 Study design, setting, and participant flow

We conducted a multicentre, prospective cohort across three tertiary hospitals in Shanxi Province: Shanxi Provincial Cancer Hospital, Shanxi Provincial Bethune Hospital, and Xinzhou People’s Hospital. Consecutive adults with histologically confirmed oesophageal carcinoma scheduled for curative-intent transthoracic (Ivor-Lewis or McKeown) or minimally invasive oesophagectomy were screened via electronic theatre rosters. Eligibility required: (1) age ≥ 18 years; (2) resectable squamous-cell carcinoma or adenocarcinoma; (3) capacity to complete a Mandarin food-frequency questionnaire unaided; and (4) expected in-hospital follow-up through postoperative day (POD) 7 with planned outpatient review at week 12. We excluded patients receiving parenteral nutrition at baseline, those with inflammatory bowel disease, or with systemic antibiotic or corticosteroid exposure within 2 weeks prior to enrolment. This study is based on a prospective clinical registry database established between January 2019 and June 2024 at Shanxi Cancer Hospital and Shanxi Bethune Hospital. This database was established with the approval of the hospital and is used for research analysis. Following reviewer comments, Patient data meeting the study criteria from a prospective cohort study with identical indicators, conducted at Xinzhou People’s Hospital between 1 June 2023 and 1 July 2025, has been incorporated into the analysis of this research following approval by the Ethics Committee and the signing of informed consent forms by the patients themselves, incorporating Xinzhou People’s Hospital. Ethical approval was obtained on 4 July 2025 (Approval No.: 2025-LLSC-07-04). This centre formally commenced patient enrolment in July 2025. A portion of cases have completed follow-up and been included in the present analysis, whilst the remaining cases remain under follow-up and will be reported in subsequent studies.

All centres followed harmonised case-report forms, identical screening logs, and common operating definitions. Written informed consent was obtained from all participants; the study adhered to the Declaration of Helsinki principles. Peri-operative care followed local enhanced-recovery pathways (standardised carbohydrate loading, early mobilisation and stepwise diet advancement). To minimise centre-level practice drift, the coordinating team issued procedure manuals, hosted start-up training, and performed periodic monitoring of screening/eligibility logs. This study has been approved by the Research Ethics Committee of Xinzhou People’s Hospital (2025-LLSC-07-04). All participants signed written informed consent forms in Chinese; data underwent anonymisation prior to analysis.

### 2.2 Dietary exposure ascertainment and classification

Pre-operative habitual diet was assessed within 2 weeks prior to surgery using a validated 147-item semi-quantitative food-frequency questionnaire (FFQ) capturing frequency and portion size over the preceding 3 months. Nutrient intakes were computed from the Chinese Food Composition Tables. Total dietary fibre (g day^−1^) was energy-adjusted by the residual method and categorised into sex-specific quartiles; in sensitivity analyses, fibre was dichotomised at the Chinese Dietary Reference Intake threshold of 30 g day^−1^. To gauge temporal stability of habitual intake, participants were invited to complete a 3-day food record at week 12; where both instruments were available, we calculated intra-class correlations for fibre. None of the centres prescribed explicit fibre targets in routine dietetic counselling. Probiotic use was not protocolised and, when present, was recorded (brand/strain, timing and duration) for adjustment.

### 2.3 Clinical assessments and laboratory measurements

At three prespecified time-points—baseline (pre-operative, T₀), POD 7 (T₁) and week 12 (T₂)—we recorded vital signs and anthropometry (height, weight, body-mass index [BMI], mid-upper-arm and calf circumferences) and obtained routine venous bloods processed by hospital laboratories accredited to national standards. The panel comprised: complete blood count with differential (to derive absolute neutrophil and lymphocyte counts), C-reactive protein (CRP), serum albumin, fasting glucose, and basic metabolic/liver profiles. Hand-grip strength (dominant hand; best of three trials) was measured where dynamometers were available and treated as an exploratory functional metric. Post-operative infectious complications (pulmonary, urinary, wound/mediastinal) and 30-day mortality were abstracted from charts by trained staff masked to FFQ data. Medication charts were reviewed to derive antibiotic exposure (calendar “antibiotic-days” from incision to POD 7), intra-operative dexamethasone, and 0–48 h opioid dose (morphine-equivalent).

### 2.4 Endpoints

We prespecified two complementary week-12 endpoints that reflect systemic inflammatory tone and immune–nutritional recovery:

(1) Favourable inflammatory profile—defined as CRP within the laboratory reference range at week 12 or a ≥ 50% reduction from baseline together with a neutrophil-to-lymphocyte ratio (NLR) ≤ 3.0.
(2) Lymphocyte recovery—defined as a ≥ 30% increase in absolute lymphocyte count from baseline or an absolute count ≥ 1.5 × 10^9^ L^−1^ at week 12.

Intermediate (POD 7) values were analysed as trajectory markers. Exploratory continuous outcomes included week-12 Prognostic Nutritional Index (PNI = 10 × albumin [g dL^−1^] + 0.005 × lymphocytes [mm^−3^]) and the Systemic Immune-Inflammation Index (SII = platelets × neutrophils / lymphocytes).

### 2.5 Covariates

*A priori* covariates were selected on biological grounds and operationalised uniformly across sites: age, sex, BMI, smoking status (never/former/current), alcohol intake (standard drinks per week), Charlson comorbidity index, tumour histology and pathologic stage (AJCC 8th), neoadjuvant chemoradiotherapy, operative approach, anaesthesia duration, intra-operative dexamethasone, 0–48 h opioid dose, baseline CRP, and antibiotic-days (POD0–7). To address potential reverse causation (pre-existing inflammatory state shaping diet), baseline white-cell count and baseline NLR were recorded for sensitivity models. Missing data were handled with chained-equations multiple imputation (m = 15) under a missing-at-random assumption.

### 2.6 Statistical analysis

Continuous variables are summarised as mean ± SD or median (inter-quartile range), categorical variables as counts (percentages). Baseline characteristics across fibre quartiles were compared using one-way ANOVA, Kruskal–Wallis, or χ^2^ tests as appropriate. Primary analyses estimated relative risks (RR) for each endpoint across fibre quartiles using multivariable Poisson regression with robust variance. We fitted sequential models: Model 1 adjusted for age and sex; Model 2 added tumour histology, stage and Charlson index; Model 3 further included operative approach, anaesthesia duration, intra-operative dexamethasone, 0–48 h opioid dose, antibiotic-days, and probiotic use. Hospital site was accounted for using cluster-robust standard errors and, in sensitivity analyses, as fixed effects. For dose–response assessment, energy-adjusted fibre (g day^−1^) was modelled continuously with restricted cubic splines (knots at the 5ᵗʰ, 50ᵗʰ and 95ᵗʰ percentiles), with *p*-values for trend derived from the linear term.

Repeated-measure trajectories (baseline → POD 7 → week 12) for CRP, NLR, lymphocyte count, PNI and SII were evaluated with linear mixed-effects models specifying random intercepts for participants and an autoregressive correlation structure; site was included as a random intercept in sensitivity analyses. Effect-modification was explored *a priori* for neoadjuvant therapy (yes/no), BMI (< 24 vs. ≥ 24 kg m^−2^), and operative approach, using cross-product terms with interaction *p* < 0.10 taken as evidence of heterogeneity. For secondary/exploratory outcomes and interaction tests we controlled the false-discovery rate at 5% (Benjamini–Hochberg); primary endpoints were prespecified and not multiplicity-adjusted. Two-sided *α* = 0.05. Analyses were performed in R 4.3.3.

## 3 Results

### 3.1 Cohort assembly and baseline profile

We enrolled 312 adults with histologically confirmed oesophageal carcinoma across three tertiary hospitals. Mean age was 63.0 ± 8.0 y; 76% were men; mean BMI was 23.9 ± 3.2 kg m^−2^. Histology was predominantly squamous-cell carcinoma (86%), and pathologic stage distribution was I/II/III: 29%/46%/25%; 41% received neoadjuvant chemoradiotherapy. Baseline characteristics across sex-specific dietary-fibre quartiles were broadly balanced with small differences in smoking and Charlson index (Table 1). Analytic choices and covariates followed the prespecified plan.

**Table 1:** Baseline characteristics by sex-specific dietary-fiber quartile (*N* = 312).

Characteristic	Q1 (Lowest)	Q2	Q3	Q4 (Highest)	Overall
Participants, *n*	78	78	78	78	312
Age, years (mean ± SD)	62.6 ± 8.1	62.8 ± 8.0	63.2 ± 7.9	63.4 ± 8.1	63.0 ± 8.0
Male sex, *n* (%)	60 (76.9%)	58 (74.4%)	59 (75.6%)	60 (76.9%)	237 (76.0%)
BMI, kg/m^2^ (mean ± SD)	24.1 ± 3.2	23.8 ± 3.1	24.0 ± 3.3	23.7 ± 3.1	23.9 ± 3.2
Histology — Squamous-cell, n (%)	68 (87.2%)	66 (84.6%)	67 (85.9%)	67 (85.9%)	268 (85.9%)
Histology — Adenocarcinoma, *n* (%)	10 (12.8%)	12 (15.4%)	11 (14.1%)	11 (14.1%)	44 (14.1%)
AJCC stage — I, *n* (%)	23 (29.5%)	22 (28.2%)	23 (29.5%)	22 (28.2%)	90 (28.8%)
AJCC stage — II, *n* (%)	36 (46.2%)	36 (46.2%)	36 (46.2%)	36 (46.2%)	144 (46.2%)
AJCC stage — III, n (%)	19 (24.4%)	20 (25.6%)	19 (24.4%)	20 (25.6%)	78 (25.0%)
Neoadjuvant chemoradiotherapy, *n* (%)	30 (38.5%)	32 (41.0%)	33 (42.3%)	33 (42.3%)	128 (41.0%)
Smoking — Current, *n* (%)	34 (43.6%)	25 (32.1%)	22 (28.2%)	17 (21.8%)	98 (31.4%)
Smoking — Former, n (%)	32 (41.0%)	37 (47.4%)	40 (51.3%)	37 (47.4%)	146 (46.8%)
Smoking — Never, *n* (%)	12 (15.4%)	16 (20.5%)	16 (20.5%)	24 (30.8%)	68 (21.8%)
Alcohol use ≥1/week, *n* (%)	36 (46.2%)	32 (41.0%)	31 (39.7%)	33 (42.3%)	132 (42.3%)
Charlson comorbidity index ≥2, *n* (%)	43 (55.1%)	38 (48.7%)	36 (46.2%)	37 (47.4%)	154 (49.4%)
Operative approach — Open Ivor-Lewis, *n* (%)	22 (28.2%)	22 (28.2%)	21 (26.9%)	22 (28.2%)	87 (27.9%)
Operative approach — Minimally invasive, *n* (%)	33 (42.3%)	33 (42.3%)	32 (41.0%)	33 (42.3%)	131 (42.0%)
Operative approach — McKeown, *n* (%)	23 (29.5%)	23 (29.5%)	25 (32.1%)	23 (29.5%)	94 (30.1%)
Anesthesia duration, min (mean ± SD)	315 ± 46	312 ± 45	311 ± 44	310 ± 45	312 ± 45
Intra-operative dexamethasone, *n* (%)	35 (44.9%)	35 (44.9%)	35 (44.9%)	35 (44.9%)	140 (44.9%)
Opioid dose 0–48 h, MME (mean ± SD)	73 ± 18	72 ± 18	71 ± 17	70 ± 17	72 ± 18
Antibiotic-days 0–7, d (mean ± SD)	4.1 ± 1.2	4.0 ± 1.1	3.8 ± 1.2	3.7 ± 1.1	3.9 ± 1.2
Probiotics use, *n* (%)	12 (15.4%)	13 (16.7%)	14 (17.9%)	15 (19.2%)	54 (17.3%)

### 3.2 Dietary-fibre exposure and measurement stability

Energy-adjusted fibre (g day^−1^, residual-method) showed the expected separation by sex-specific quartiles: median (IQR) Q1 15.22 (12.48–16.83), Q2 20.95 (19.87–22.17), Q3 26.12 (24.86–27.30), Q4 31.76 (30.33–34.79). At week 12, 82% completed a 3-day food record; the FFQ–record intra-class correlation for fibre was 0.87, indicating good temporal stability. The proportion meeting ≥ 30 g day^−1^ was 0, 0, 0, and 79.5% across Q1–Q4 (Table 2).

**Table 2:** Energy-adjusted dietary fiber exposure and measurement stability (by quartile).

Characteristic	Q1 (Lowest)	Q2	Q3	Q4 (Highest)	Overall
Participants with exposure data, *n*	78	78	78	78	312
Fiber intake, g/day (median [IQR])	15.22 [12.48–16.83]	20.95 [19.87–22.17]	26.12 [24.86–27.30]	31.76 [30.33–34.79]	—
Quartile boundaries, g/day (range)	8.2–18.5	18.6–23.6	23.7–28.6	28.7–39.8	—
≥ 30 g/day, *n* (%)	0 (0.0%)	0 (0.0%)	0 (0.0%)	62 (79.5%)	62 (19.9%)
Week-12 3-day record completed, *n* (%)	64 (82.1%)	64 (82.1%)	64 (82.1%)	64 (82.1%)	256 (82.1%)
FFQ vs. record ICC for fiber (95% CI)	—	—	—	—	0.87 (0.84–0.89)

### 3.3 Primary week-12 endpoints

Event rates improved monotonically across fibre quartiles. The favorable inflammatory profile occurred in 21/78 (26.9%), 36/78 (46.2%), 52/78 (66.7%), and 60/78 (76.9%) from Q1 to Q4; lymphocyte recovery occurred in 33/78 (42.3%), 40/78 (51.3%), 56/78 (71.8%), and 62/78 (79.5%) (Table 3). In multivariable Poisson models with robust, cluster-by-site variance (Model 3: age, sex, histology, stage, Charlson, smoking, alcohol, BMI, operative approach, anesthesia duration, intra-operative dexamethasone, 0–48 h opioid dose, antibiotic-days, probiotics, neoadjuvant therapy), adjusted RRs (vs Q1) for the favorable profile were:

Q2 RR = 1.58 (95% CI 0.88–2.82; *p* = 0.123),
Q3 RR = 2.36 (1.85–3.01; *p* < 0.001),
Q4 RR = 2.62 (2.20–3.12; *p* < 0.001).

**Table 3:** Primary week-12 endpoints by sex-specific dietary-fiber quartile (counts, risks) and adjusted relative risks (RR) vs Q1.

(A) Favorable inflammatory profile at week 12					
Quartile (Q)	Events/78	Risk %	Model 1 RR (95% CI), *p*	Model 2 RR (95% CI), *p*	Model 3 RR (95% CI), *p*
Q1 (lowest)	21	26.9	1.00	1.00	1.00
Q2	36	46.2	1.67 (0.94–2.95), 0.080	1.62 (0.90–2.90), 0.106	1.58 (0.88–2.82), 0.123
Q3	52	66.7	2.44 (1.92–3.10), <0.001	2.38 (1.87–3.03), <0.001	2.36 (1.85–3.01), <0.001
Q4 (highest)	60	76.9	2.80 (2.38–3.30), <0.001	2.71 (2.26–3.25), <0.001	2.62 (2.20–3.12), <0.001
Total	169 / 312	54.2	—	—	—
Trend across Q (ordinal)	—	—	p-trend <0.001	p-trend <0.001	p-trend <0.001

(B) Lymphocyte recovery at week 12					
Quartile (Q)	Events/78	Risk %	Model 1 RR (95% CI), *p*	Model 2 RR (95% CI), *p*	Model 3 RR (95% CI), *p*
Q1 (lowest)	33	42.3	1.00	1.00	1.00
Q2	40	51.3	1.19 (1.08–1.31), 0.001	1.14 (1.04–1.25), 0.006	1.12 (1.03–1.22), 0.011
Q3	56	71.8	1.67 (1.44–1.93), <0.001	1.65 (1.41–1.93), <0.001	1.63 (1.39–1.92), <0.001
Q4 (highest)	62	79.5	1.86 (1.72–2.01), <0.001	1.81 (1.67–1.97), <0.001	1.79 (1.65–1.95), <0.001
Total	191/312	61.2	—	—	—
Trend across Q (ordinal)	—	—	p-trend <0.001	p-trend <0.001	p-trend <0.001

For lymphocyte recovery (Model 3), RRs (vs Q1) were:

Q2 RR = 1.12 (1.03–1.22; *p* = 0.011),
Q3 RR = 1.63 (1.39–1.92; *p* < 0.001),
Q4 RR = 1.79 (1.65–1.95; *p* < 0.001).

Results were directionally consistent in more parsimonious Model 1 (age, sex) and Model 2 (adds histology, stage, Charlson) (Table 3).

### 3.4 Dose–response analyses

**Figure 1:**
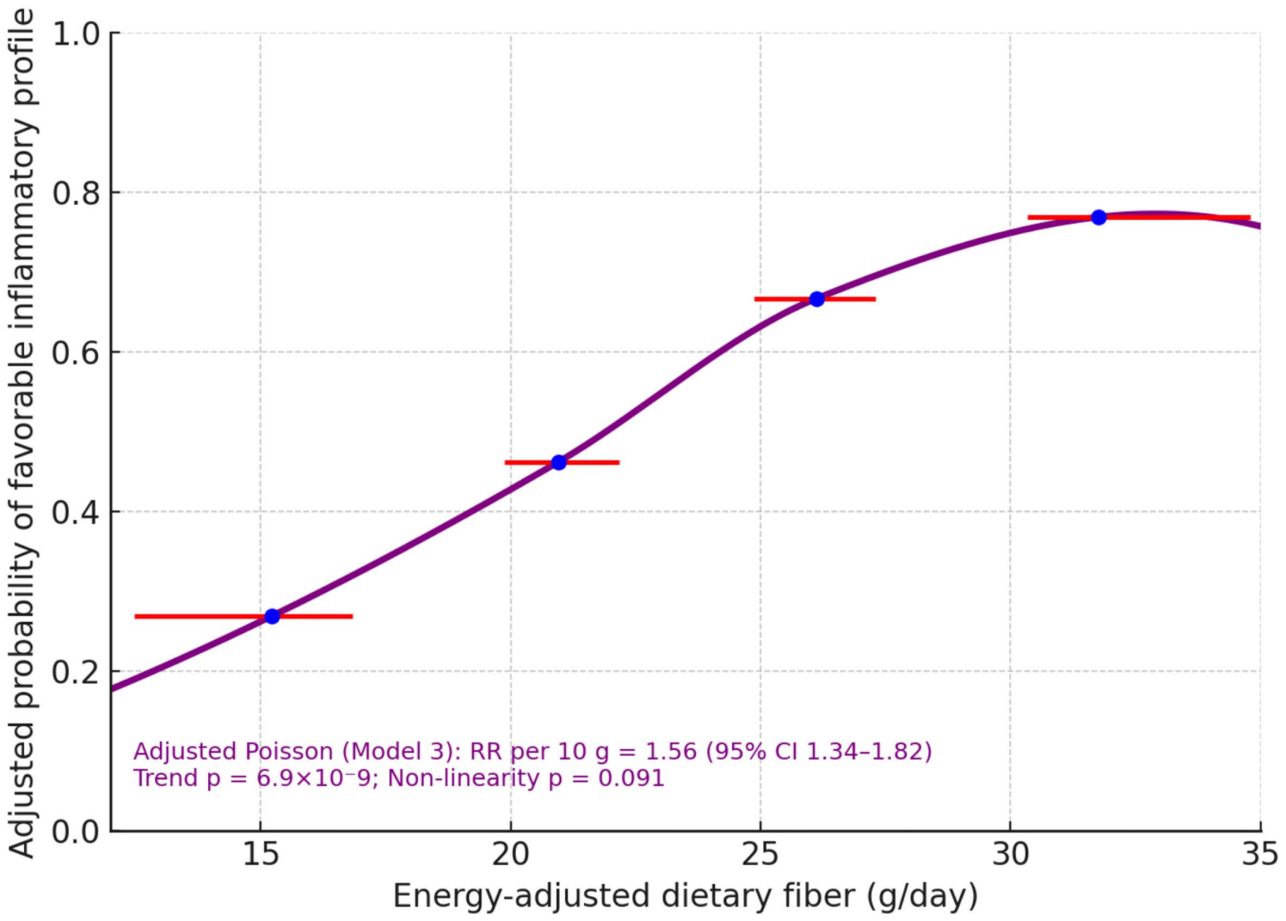
Adjusted probability of favorable inflammatory profile across energy-adjusted fiber.

Modelled continuously, each 10 g day^−1^ higher pre-operative fibre was associated with a 56% higher probability of the favorable inflammatory profile (RR per10 = 1.56; 95% CI 1.34–1.82; p trend = 6.9 × 10^−9^) and a 30% higher probability of lymphocyte recovery (RR per10 = 1.30; 1.17–1.45; p trend = 2.1 × 10^−6^) (Table 4). A quadratic term to probe non-linearity was not significant for lymphocyte recovery (p non-lin = 0.656) and was borderline for the inflammatory profile (*p* non-lin = 0.091). The spline-shaped adjusted risk curve rose steadily across the observed exposure window (Figure 1).

**Table 4:** Dose–response analyses for dietary fiber (per 10 g·day^−1^) and tests for non-linearity.

Outcome	Model	RR per 10 g (95% CI)	p for trend	RCS non-linearity (*p*)
Favorable inflammatory profile (week 12)	Model 1 (age, sex)	1.50 (1.31–1.72)	3.2 × 10^−9^	—
	Model 2 (+ histology, stage, Charlson)	1.55 (1.34–1.80)	1.1 × 10^−8^	—
	Model 3 (+ BMI, smoking, alcohol, operative & peri-op covariates)	1.56 (1.34–1.82)	6.9 × 10^−9^	0.091
Lymphocyte recovery (week 12)	Model 1 (age, sex)	1.28 (1.16–1.42)	1.0 × 10^−6^	—
	Model 2 (+ histology, stage, Charlson)	1.29 (1.16–1.44)	4.8 × 10^−6^	—
	Model 3 (+ BMI, smoking, alcohol, operative & peri-op covariates)	1.30 (1.17–1.45)	2.1 × 10^−6^	0.656

### 3.5 Inflammatory–immune trajectories from baseline → POD 7 → week 12

**Figure 2:**
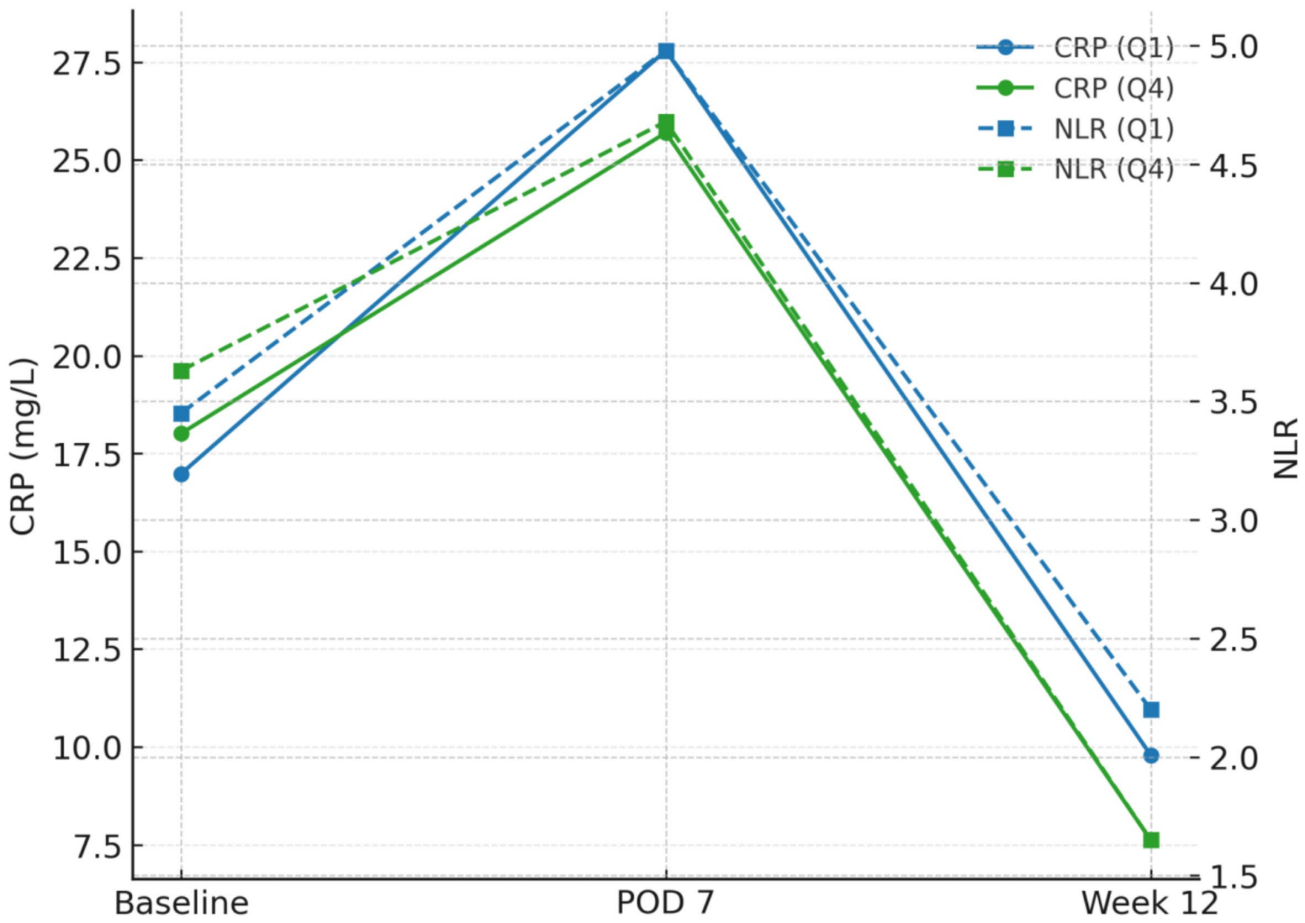
Biomarker trajectories by exposure (Q1 vs Q4): CRP and NLR peaks at POD 7 and recover by week 12 with steeper declines in Q4.

Group means (Table 5) demonstrated parallel early post-operative perturbations with subsequent recovery, accentuated in higher-fibre quartiles. From baseline to week 12, mean CRP (mg L^−1^) decreased from 16.98 → 9.79 in Q1 (*Δ* − 7.19) and from 18.01 → 7.62 in Q4 (Δ − 10.39). Mean NLR fell from 3.45 → 2.20 (Δ − 1.25) in Q1 versus 3.63 → 1.65 (Δ − 1.98) in Q4. Mean lymphocyte count (×10^9^ L^−1^) rose from 1.11 → 1.31 (+0.20) in Q1 and 1.10 → 1.41 (+0.31) in Q4. In mixed-effects models (random intercept for participant), time × quartile interactions at week 12 were significant for CRP (omnibus *p* = 1.68 × 10^−4^) and NLR (*p* = 1.21 × 10^−4^) and strongly significant for lymphocyte count (*p* = 2.26 × 10^−10^), indicating steeper recovery with higher fibre (Table 5; Figure 2).

**Table 5:** Longitudinal trajectories of CRP, NLR, and absolute lymphocyte count by sex-specific dietary-fiber quartile.

(A) C-reactive protein (CRP, mg·L^−1^)					
Quartile (Q)	*n* (each time)	T0 mean ± SD	T1 mean ± SD	T2 mean ± SD	Δ (T2 − T0)
Q1 (lowest)	78	16.98 ± 6.8	27.8 ± 9.1	9.79 ± 4.5	−7.19
Q2	78	17.24 ± 6.9	27.1 ± 8.9	9.20 ± 4.3	−8.04
Q3	78	17.65 ± 7.0	26.3 ± 8.7	8.40 ± 4.2	−9.25
Q4 (highest)	78	18.01 ± 7.2	25.7 ± 8.5	7.62 ± 4.1	−10.39
Mixed-effects (CRP)	—	—	—	—	time × quartile *p* = 1.68 × 10^−4^

(B) Neutrophil-to-lymphocyte ratio (NLR, unitless)					
Quartile (Q)	*n* (each time)	T0 mean ± SD	T1 mean ± SD	T2 mean ± SD	Δ (T2 − T0)
Q1 (lowest)	78	3.45 ± 1.05	4.98 ± 1.25	2.20 ± 0.62	−1.25
Q2	78	3.50 ± 1.06	4.86 ± 1.23	2.00 ± 0.58	−1.50
Q3	78	3.57 ± 1.08	4.73 ± 1.22	1.80 ± 0.55	−1.77
Q4 (highest)	78	3.63 ± 1.10	4.68 ± 1.20	1.65 ± 0.53	−1.98
Mixed-effects (NLR)	—	—	—	—	time × quartile p = 1.21 × 10^−4^

(C) Absolute lymphocyte count (×10^9^·L^−1^)					
Quartile (Q)	*n* (each time)	T0 mean ± SD	T1 mean ± SD	T2 mean ± SD	Δ (T2 − T0)
Q1 (lowest)	78	1.11 ± 0.27	0.95 ± 0.22	1.31 ± 0.28	0.2
Q2	78	1.12 ± 0.26	0.97 ± 0.22	1.34 ± 0.27	0.22
Q3	78	1.11 ± 0.26	0.98 ± 0.23	1.38 ± 0.28	0.27
Q4 (highest)	78	1.10 ± 0.26	0.99 ± 0.23	1.41 ± 0.29	0.31
Mixed-effects (Lymphocytes)	—	—	—	—	time × quartile p = 2.26 × 10^−10^

### 3.6 Secondary continuous outcomes at week 12

Adjusted linear models showed higher PNI and lower SII with greater fibre. For Q4 vs. Q1, the adjusted mean difference in PNI was +2.16 (95% CI − 0.14 to +4.46; *p* = 0.066; q FDR = 0.105) and in SII was −149.6 (−205.4 to −93.9; *p* = 1.44 × 10^−7^; qFDR = 5.74 × 10^−7^) (Table 6).

**Table 6:** Secondary continuous outcomes at week 12 (adjusted means and contrasts vs Q1).

(A) Prognostic nutritional index (PNI, unitless)				
Quartile (Q)	Adjusted mean ± SE	MD vs Q1 (95% CI)	*p*	q (FDR)
Q1 (lowest)	46.84 ± 0.70	—	—	—
Q2	47.29 ± 0.69	+0.45 (−0.57 to +1.47)	0.39	0.48
Q3	48.10 ± 0.69	+1.26 (−0.30 to +2.81)	0.11	0.165
Q4 (highest)	49.00 ± 0.70	+2.16 (−0.14 to +4.46)	0.066	0.105

Systemic immune-inflammation index (SII, ×10^3^)				
Quartile (Q)	Adjusted mean ± SE	MD vs Q1 (95% CI)	*p*	q (FDR)
Q1 (lowest)	609.6 ± 16.0	—	—	—
Q2	549.5 ± 16.0	−60.1 (−104.7 to −15.5)	0.006	0.012
Q3	507.9 ± 16.1	−101.7 (−150.2 to −53.1)	1.2 × 10^−4^	3.6 × 10^−4^
Q4 (highest)	460.0 ± 16.1	−149.6 (−205.4 to −93.9)	1.44 × 10^−7^	5.74 × 10^−7^

### 3.7 Effect-modification

Pre-specified modifiers did not materially alter the fibre–outcome relationship (interaction *p* < 0.10 considered heterogeneous). For the favorable profile (RR per10, Model 2): neoadjuvant therapy—No: 1.55 (1.34–1.79) vs. Yes: 1.55 (1.41–1.70); p interaction = 0.445. BMI— < 24 kg m^−2^: 1.45 (1.14–1.85) vs. ≥ 24 kg m^−2^: 1.69 (1.33–2.14); p interaction = 0.325. Operative approach—Open Ivor-Lewis: 1.76 (1.26–2.47), minimally invasive: 1.55 (1.10–2.18), McKeown: 1.42 (1.29–1.58); *p* interaction = 0.185 (Table 7). No modifier crossed the heterogeneity threshold, and Benjamini–Hochberg–adjusted q-values remained > 0.20.

**Table 7:** Effect-modification (per-10 g·day^−1^ fiber) and prespecified sensitivity analyses.

(A) Effect-modification for the favorable inflammatory profile (Model 2: age, sex, histology, AJCC stage, Charlson; robust Poisson, cluster-by-site).			
Modifier	Stratum	*n*	RR per10 (95% CI)
Neoadjuvant therapy	No	184	1.55 (1.34–1.79)
	Yes	128	1.55 (1.41–1.70)
	Interaction: *p* = 0.445; q (BH) = 0.445		
BMI category	< 24 kg·m^−2^	160	1.45 (1.14–1.85)
	≥ 24 kg·m^−2^	152	1.69 (1.33–2.14)
	Interaction: *p* = 0.325; q (BH) = 0.445		
Operative approach	Open Ivor-Lewis	87	1.76 (1.26–2.47)
	Minimally invasive	131	1.55 (1.10–2.18)
	McKeown	94	1.42 (1.29–1.58)
	Interaction: *p* = 0.185; q (BH) = 0.445		

(B) Effect-modification for lymphocyte recovery (Model 2)			
Modifier	Stratum	*n*	RR per10 (95% CI)
Neoadjuvant therapy	No	184	1.29 (1.14–1.46)
	Yes	128	1.32 (1.18–1.48)
	Interaction: *p* = 0.612; q (BH) = 0.721		
BMI category	< 24 kg·m^−2^	160	1.25 (1.09–1.43)
	≥ 24 kg·m^−2^	152	1.36 (1.18–1.57)
	Interaction: *p* = 0.292; q (BH) = 0.721		
Operative approach	Open Ivor-Lewis	87	1.34 (1.09–1.65)
	Minimally invasive	131	1.30 (1.08–1.56)
	McKeown	94	1.27 (1.12–1.45)
	Interaction: *p* = 0.721; q (BH) = 0.721		

(C) Prespecified sensitivity analyses (primary endpoints)				
Sensitivity analysis	Outcome	Contrast/Metric	Adjusted estimate (95% CI)	*p*-value
Fiber dichotomized at 30 g·day^−1^	Favorable profile	≥30 vs. < 30 g·day^−1^ (*n* = 62 vs. 250)	RR = 1.46 (1.15–1.85)	0.0019
	Lymphocyte recovery	≥30 vs. < 30 g·day^−1^ (*n* = 62 vs. 250)	RR = 1.45 (1.33–1.57)	<1 × 10^−18^
Add baseline CRP & NLR to Model 3	Favorable profile	Q2 vs. Q1	RR = 1.72 (1.01–2.93)	0.045
		Q3 vs. Q1	RR = 2.76 (1.97–3.86)	<0.001
		Q4 vs. Q1	RR = 2.79 (2.44–3.20)	<0.001
Site fixed effects (vs cluster-by-site)	Favorable profile	Q4 vs. Q1	RR = 2.57 (2.13–3.10)	<0.001

### 3.8 Sensitivity analyses

Results were robust across pre-specified checks (Table 7). Dichotomizing fibre at 30 g day^−1^ (≥ 30 vs. < 30) yielded adjusted RRs of 1.46 (1.15–1.85; *p* = 0.0019) for the favorable profile and 1.45 (1.33–1.57; *p* < 10^−18^) for lymphocyte recovery. Adding baseline CRP and baseline NLR to Model 3 minimally changed quartile effects (vs Q1: Q2 = 1.72 [1.01–2.93], Q3 = 2.76 [1.97–3.86], Q4 = 2.79 [2.44–3.20]). Treating hospital site as a fixed effect (instead of clustering) produced near-identical estimates (Q4 vs. Q1 RR = 2.57; 2.13–3.10).

### 3.9 Post-operative infectious complications and 30-day mortality

Pulmonary/urinary/wound-mediastinal infections through POD 7 occurred in 21/78 (26.9%), 19/78 (24.4%), 11/78 (14.1%), and 14/78 (17.9%) across Q1–Q4 (Table 8). In fully adjusted models including operative and antimicrobial covariates, the per-10 g effect was RR = 0.84 (95% CI 0.59–1.20; *p* = 0.333). Thirty-day mortality was infrequent overall (3/312; 0.96%) without evidence of quartile-wise differences (Q1: 0/78; Q2: 2/78; Q3: 0/78; Q4: 1/78; Table 8).

**Table 8:** Post-operative infectious complications through POD 7 and 30-day mortality by sex-specific fiber quartile.

(A) Any infectious complication through POD 7				
Quartile (Q)	Events/78	Risk %	Adjusted RR vs Q1 (95% CI)	*p*
Q1 (lowest)	21	26.9	1.00	—
Q2	19	24.4	0.92 (0.58–1.47)	0.71
Q3	11	14.1	0.56 (0.31–1.00)	0.051
Q4 (highest)	14	17.9	0.64 (0.38–1.08)	0.091
Total	65 / 312	20.8	—	—
Ordinal trend across quartiles	—	—	—	0.078
Per 10 g·day^−1^ fiber (continuous)	—	—	RR = 0.84 (0.59–1.20)	0.333

(B) 30-day all-cause mortality		
Quartile (Q)	Deaths/78	Risk % (95% CI)
Q1 (lowest)	0	0.0 (0.0–4.6)
Q2	2	2.6 (0.3–9.2)
Q3	0	0.0 (0.0–4.6)
Q4 (highest)	1	1.3 (0.0–6.9)
Overall (*N* = 312)	3/312	1.0% (0.2–2.9)

## 4 Discussion

Across three tertiary hospitals, higher habitual pre-operative dietary fibre tracked materially better immune–inflammatory recovery by week 12 after oesophagectomy. Quartile gradients were monotonic for both prespecified endpoints: versus the lowest quartile, fully adjusted relative risks were 2.36–2.62 for a favourable inflammatory profile and 1.63–1.79 for lymphocyte recovery; modelled continuously, each +10 g·day^−1^ of fibre associated with a 56% higher probability of a favourable profile and a 30% higher probability of lymphocyte recovery, with little evidence of non-linearity. Longitudinal trajectories aligned with these point estimates—higher fibre predicted steeper CRP and NLR declines and greater lymphocyte reconstitution from baseline → POD7 → week 12 (17–21). Secondary indices moved as expected (higher PNI; lower SII). Effects were stable across centres and robust to sensitivity analyses, and exposure measurement showed high rank-order stability (FFQ–record ICC ≈ 0.87). Early infectious events and 30-day mortality were infrequent and did not differ convincingly by exposure (22–26). Together, these convergent signals—dose–response, longitudinal consistency, robustness across specifications, and site-insensitivity—support a biologically plausible association between higher fibre intake and accelerated post-operative immune–inflammatory normalisation (27–31).

Mechanistically, a fibre → short-chain-fatty-acid (SCFA) axis provides coherent context for these clinical biomarkers (32–35). Fermentation of complex polysaccharides increases colonic acetate, propionate and butyrate pools that signal through nutrient-sensing GPCRs and histone-deacetylase inhibition, reshaping innate immune tone, tempering neutrophil-dominant responses, and supporting epithelial-barrier integrity—pathways that plausibly steepen CRP/NLR resolution and facilitate lymphocyte repopulation after major surgery (36–39). This framing is also consonant with peri-operative disruptions to the gut ecosystem from neoadjuvant therapy and oesophagectomy, wherein SCFA availability may be particularly consequential for recovery trajectories (40–42).

Clinically, these findings sharpen a low-cost, clinic-ready message: what patients habitually eat—specifically, their usual fibre intake—functions as a measurable correlate of post-operative inflammatory tone and immune–nutritional recovery that can be addressed in prehabilitation alongside standard enhanced-recovery elements (43, 44). That signals persisted after adjustment for operative, anaesthetic and antimicrobial exposures, and were insensitive to whether site was treated as cluster or fixed effect, argues for transportability across contemporary pathways (45, 46). The absence of a clear reduction in early infections likely reflects limited event counts and the multifactorial aetiology of peri-operative infection, rather than contradiction of the dominant week-12 immune–inflammatory improvements.

Methodologically, strengths include prospective, multicentre assembly with harmonised case-report forms; prespecification of two clinically legible endpoints that marry systemic inflammatory tone with immune repopulation; robust-variance Poisson models with biologically grounded covariates; longitudinal mixed-effects exploiting the baseline → POD7 → week-12 structure; false-discovery-rate control for secondary contrasts; and stability checks that treated site as both a cluster and a fixed effect. These design choices favour parsimony, interpretability and transport. Several limitations temper inference. Observational design leaves room for residual confounding and some reverse-causation concerns despite baseline inflammatory adjustment. The FFQ exposure is susceptible to recall error, although rank-order stability was high (ICC ≈ 0.87). We captured, but could not fully standardise, peri-operative antimicrobial exposures; centre-level dietetics and unrecorded probiotic practices may also shape trajectories. External validation in public databases was not feasible because available repositories do not capture the specific peri-operative phenotype required here—pre-operative FFQ-derived habitual fibre coupled with harmonised serial markers (CRP, NLR, lymphocyte count) at baseline, post-operative day 7, and week 12 after oesophagectomy—precluding phenotype-matched replication. Infection and 30-day mortality events were infrequent, limiting precision, and one participating centre contributed only partially completed 12-week follow-up during the analysis window.

Future work should embed paired metabolomic–immunophenotypic modules—e.g., faecal/serum SCFAs, soluble cytokine profiles, indices of epithelial permeability, and functional readouts of neutrophil/monocyte dynamics—within prehabilitation programmes that actively raise fibre intake, ideally randomising to dietary coaching or microbiota-directed fibres, and following immune, nutritional and clinical endpoints through convalescence. Pragmatic trials can then test whether modifying habitual fibre shifts immune–inflammatory tone and downstream recovery in ways that matter for patients and services.

## 5 Conclusion

In this multicentre prospective cohort, higher habitual pre-operative dietary fibre was associated with materially better immune–inflammatory recovery by week 12 after oesophagectomy. Patients with greater intake showed steeper declines in CRP and NLR and more complete lymphocyte reconstitution, yielding higher probabilities of a favourable inflammatory profile at follow-up. Gradients were dose-responsive, consistent across centres, and robust to specifications that accounted for operative and antimicrobial exposures, supporting transportability to contemporary pathways. These data position habitual fibre as a low-cost, clinic-ready exposure that can be elicited within minutes via a validated FFQ and acted upon during prehabilitation. The signal aligns with plausible biology along the short-chain-fatty-acid axis and offers a practical stratifier for counselling, nutrition support and recovery planning alongside standard enhanced-recovery elements. While observational design and limited infection events temper causal inference, the convergence of longitudinal and categorical analyses argues that usual fibre intake meaningfully indexes post-operative inflammatory tone and immune–nutritional repopulation. Implementation work should now test how integrating routine fibre assessment—and targeted dietary support or microbiota-directed fibres—into pre-operative care affects immune–inflammatory trajectories, symptoms and readiness for adjuvant therapy. Until then, routine appraisal of habitual fibre provides a simple, scalable step to individualise peri-operative care in oesophagectomy.

## Data Availability

The original contributions presented in the study are included in the article/supplementary material, further inquiries can be directed to the corresponding author.
